# The SFT-1 and OXA-1 respiratory chain complex assembly factors influence lifespan by distinct mechanisms in *C. elegans*

**DOI:** 10.1186/2046-2395-2-9

**Published:** 2013-05-08

**Authors:** Sara Maxwell, Joanne Harding, Charles Brabin, Peter J Appleford, Ruth Brown, Carol Delaney, Garry Brown, Alison Woollard

**Affiliations:** 1 Biochemistry Department, University of Oxford, South Parks Road, Oxford, OX1 3QU, UK; 2 Present address: CRUK London Research Institute, 44 Lincoln’s Inn Fields, London, WC2A 3LY, UK; 3 Present address: Cytogenetics Department, South East Scotland Genetics Service, Western General Hospital, Edinburgh, UK

**Keywords:** Mitochondria, Respiratory chain complex, Aging, Lifespan, *C. elegans*, SURF1, OXA-1

## Abstract

**Background:**

*C. elegans* mitochondrial (Mit) mutants have disrupted mitochondrial electron transport chain function, yet, surprisingly, they are often long-lived, a property that has offered unique insights into the molecular mechanisms of aging. In this study, we examine the phenotypic consequences of reducing the expression of the respiratory chain complex assembly factors *sft-1* (homologous to human *SURF1*) and *oxa-1* (homologous to human *OXA1)* by RNA interference (RNAi). Mutations in human *SURF1* are associated with Leigh syndrome, a neurodegenerative condition of the brain caused by cytochrome oxidase (COX) deficiency. Both SURF1 and OXA1 are integral proteins of the inner mitochondrial membrane, functioning in the COX assembly pathway.

**Results:**

RNAi of both of these genes in *C. elegans* is associated with increased longevity, but the mechanism by which lifespan is extended is different in each case. *sft-1(RNAi)* animals display lifespan extension that is dependent on the *daf-16* insulin-like signaling pathway, and associated with sensitivity to oxidative stress. *oxa-1(RNAi)* animals, in contrast, exhibit increased longevity that is at least partially independent of *daf-16*, and associated with a reduced developmental rate and increased resistance to oxidative stress.

**Conclusions:**

This study further delineates the consequences of mitochondrial dysfunction within a whole organism that will ultimately help provide new models for human mitochondrial-associated diseases. The difference in phenotype observed upon down-regulation of these two COX assembly factors, as well as phenotypic differences between these factors and other respiratory chain components analyzed thus far, illustrates the complex inter-relationships that exist among energy metabolism, reproduction and aging even in this simplest of metazoan model organisms.

## Background

Defects in mitochondrial function are implicated in a wide range of human diseases, affecting both development and the maintenance of normal structure and function [1]. The complexes of the mitochondrial respiratory chain are each composed of multiple subunits derived from both nuclear and mitochondrial genes. Because of the dual genetic contributions, it has proved difficult to generate model systems in which to study the consequences of specific mitochondrial defects, particularly those due to primary mutations in mitochondrial DNA (mtDNA). One way to approach this problem is to exploit the observations that a number of accessory proteins are required for the assembly of respiratory chain complexes in the inner mitochondrial membrane [2-4]. Deficiencies of these complexes, either singly or in combination, can be generated by targeted disruption of the nuclear genes for these assembly factors. In addition, defects in the assembly proteins often do not result in complete deficiency of the respiratory complexes and this is an advantage when studying key components of the main energy generating system of the cell, where complete deficiency could result in early embryonic lethality.

The nematode worm, *C. elegans* is an established model organism for the study of pathogenesis of human mitochondrial diseases, exploiting the remarkable degree of evolutionary homology between both nuclear-encoded and mtDNA-encoded components of complexes of the respiratory chain, as well as associated assembly factors (reviewed in [5,6]. There are a number of reports of the consequences of defects in mitochondrial function in *C. elegans* (reviewed in [7,8]. These studies were often undertaken in the first instance to evaluate *C. elegans* as a potential model system for the investigation of pathogenic mechanisms in human mitochondrial disease; however, some unexpected phenotypic consequences of mitochondrial enzyme deficiencies were soon identified, particularly in relation to lifespan. While these are not immediately relevant to the human diseases, they may provide unique insights into the molecular mechanisms of aging [8,9].

One of the most striking findings is that different mitochondrial defects, which result in comparable levels of impairment of energy generation, can have opposite effects on lifespan. For example, mutation in the *isp-1* gene, encoding a Reiske iron sulphur protein that is a component of mitochondrial complex III, displays decreased mitochondrial respiration and significantly prolonged lifespan [10]. Likewise, *clk-1* mutants, lacking an enzyme required for ubiquinone synthesis (essential for complex I-dependent respiration), *lrs-2* mutants (lacking a mitochondrial tRNA synthetase), and *nuo-1* and *atp-2* mutants, containing defects in mitochondrial proteins affecting complexes I and V, respectively, all display an increased lifespan [11-13]. Furthermore, various genome-wide RNAi screens have identified a wide range of mitochondrial genes that, when silenced, prolong lifespan [10,12,14,15].

In contrast, the complex I subunit mutant *gas-1(fc210)*, has a shortened lifespan [16], as has the complex II subunit mutant *mev-1(kn1)* and the complex III subunit mutant *ucr-2.3(pk732)*[9,17]. Thus, there is no simple relationship between the respiratory chain complex targeted and the phenotypic outcome. An early assumption in the field, based on the “free radical theory of ageing” was that oxidative stress would play a key role in the altered longevity of mitochondrial mutants, with impaired mitochondrial function lowering the level of reactive oxygen species (ROS) and, hence, increasing lifespan. Alternatively, interfering with the function of respiratory chain complexes can in some situations lead to increased ROS and, therefore, shorten lifespan. However, extensive investigations have collectively revealed that oxidative damage can be experimentally dissociated from aging (reviewed in [8]). For example, slow physiological rates and prolonged lifespan were not found to be associated with increased stress resistance or reduced oxidative damage in Clk mutants that displayed mitochondrial dysfunction and evidence of decreased energy utilization [18]. Furthermore, oxidative stress was shown to actually increase worm lifespan in particular experimental conditions [19-22] and it has been proposed that increased ROS levels can lengthen lifespan via increased HIF-1 activity (reviewed in [23]), or by signaling to other endogenous defense mechanisms as part of an adaptive response (reviewed in [24]. Thus, it will be necessary to compare the functions of many different mitochondrial components, identifying common and conflicting features, to determine key elements in the relationship between mitochondria and aging.

In this study, we compare the consequences of the deficiency of two mitochondrial respiratory chain assembly factors, the protein products of the *sft-1* and *oxa-1* genes. Targeted silencing of these *C. elegans* genes by RNA interference (RNAi) produces model deficiencies of these proteins in a tractable system, where the phenotypic consequences can be easily defined. The gene *sft-1* is the *C. elegans* ortholog of the human *SURF1* gene. Mutations in *SURF1* result in a systemic cytochrome oxidase deficiency; however, the mutant phenotype is restricted to the brain. Patients typically present with Leigh syndrome, a subacute neurodegenerative condition with characteristic necrotic lesions of the basal ganglia and brain stem [3,25]. The *SURF1* gene product is an integral protein of the inner mitochondrial membrane, part of a 250 kDa complex which appears to act early in the COX assembly pathway [26]. Patients with SURF1p deficiency do have some residual COX activity (5 to 10%) indicating that some complex assembly can occur in the absence of this protein.

The human *OXA1* and *C. elegans oxa-1* genes encode the protein OXA1, a ubiquitous component of the inner mitochondrial membrane that inserts mitochondrially synthesized cytochrome oxidase subunits COX1, COX2 and COX3 into the inner membrane co-translationally, via binding of its C-terminal domain to the mitochondrial ribosome [27]. Human OXA1 deficiency may be embryonic lethal, as no patients have yet been described.

Although both SURF1/SFT-1 and OXA-1 are components of the inner mitochondrial membrane that function in the assembly of respiratory chain complexes, the consequences of *sft-1* and *oxa-1* knockdown in *C. elegans* are quite different. The overall result of knockdown of either gene is increased lifespan, but the mechanism by which prolonged lifespan is conferred, in terms of resistance to oxidative stress and dependence on the DAF-16/FoxO insulin-like signaling pathway component, are different, suggesting that distinct molecular pathways are involved.

## Results

### Decreased brood size in *sft-1(RNAi)* and *oxa-1(RNAi)* worms

**Figure 1:**
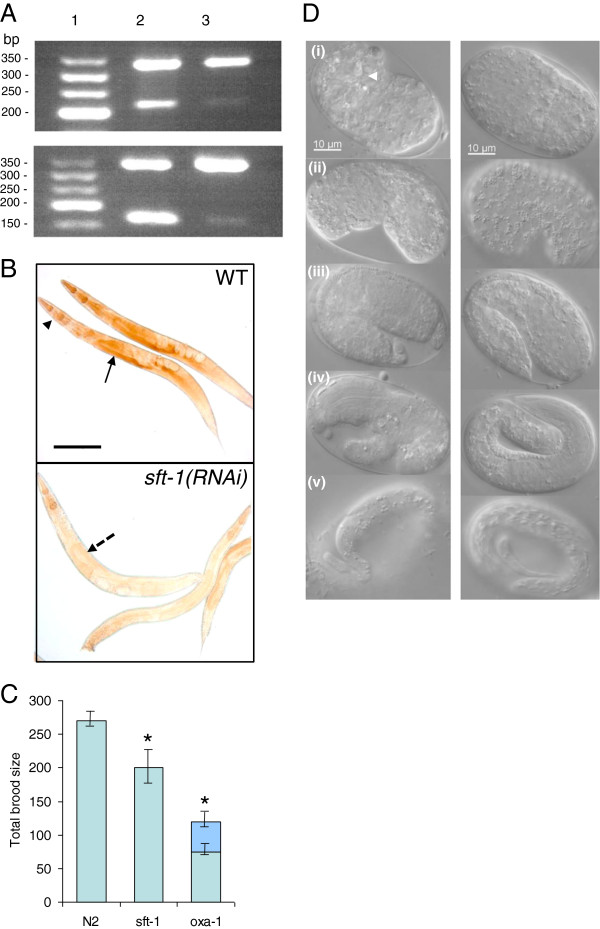
***sft-1 *or *oxa-1 *RNAi results in reduced gene expression, COX staining and brood size. A**: RT-PCR of *oxa-1* and *sft-1* to compare mRNA levels. Lane 1, 50 bp DNA ladder. Upper panel: Lane 2, RT-PCR products from WT animals. Upper band (350 bp) corresponds to *eft-2* mRNA (internal control), lower band (220 bp) corresponds to *sft-1* mRNA. Lane 3, RT-PCR products from the progeny of animals injected with *sft-1* dsRNA. *sft-1* expression is greatly reduced. Lower panel: Lane 2, RT-PCR products from WT animals. Upper band (350 bp) corresponds to the *eft-2* internal control, lower band (164 bp) corresponds to *oxa-1* mRNA. Lane 3, RT-PCR products from the progeny of animals injected with *oxa-1* dsRNA. *oxa-1* expression is greatly reduced. **B**: COX staining in WT and *sft-1(RNAi)* animals. In WT, COX staining is particularly prevalent in the pharynx (black arrowhead) and germ line (black arrow), tissues that have high energy demands. In *sft-1(RNAi*) animals, COX staining is greatly reduced (photomicrographs taken using identical exposure times), especially in the germ line (dotted arrow). Scale bar, 100 μm. **C**: Brood size of WT animals injected with *sft-1* or *oxa-1* dsRNA compared with control animals injected with TE only (labeled N2). Both *sft-1* and *oxa-1* RNAi result in a significantly reduced brood size compared with controls (*P <0.005). The darker shaded area of the bar corresponding to *oxa-1(RNAi)* represents the proportion of dead embryos. Error bars denote the standard error of the mean (SEM). **D**: Embryonic lethality induced by *oxa-1* RNAi. Left hand panel: *oxa-1(RNAi)* embryos, right hand panel: WT embryos at equivalent stages. Defects can be seen throughout embryogenesis, including necrotic regions in early embryos (i, white arrowhead), morphological defects at the “bean” stage (ii), elongation defects at the “comma” stage (iii) and later (iv, v). Posterior defects are particularly pronounced.

*sft-1* (gene *H06I04.2*) and *oxa-1* (gene *C01A2.3*) were identified as the closest *C. elegans* homologues of human *SURF1* and *OXA1L*, respectively. *sft-1* and *SURF1* both contain a Surfeit Locus 1 domain of approximately 300 amino acids, displaying 34.1% identity (53.8% similarity). In *C. elegans*, *sft-1* is represented by the single deletion allele *ok2277*. Human and *C. elegans oxa1* genes both contain a 60 kDa inner membrane protein domain of approximately 200 amino acids, displaying 21% identity (43% similarity). RNAi of *sft-1* or *oxa-1* leads to significantly reduced gene expression and consequent reduction in cytochrome oxidase activity as assayed in whole animals in the case of *sft-1* RNAi (Figure 1A,B). Knockdown of either gene was associated with decreased brood size (a 27% decrease, on average, following *sft-1* RNAi by injection, and a 57% decrease, on average, in worms injected with *oxa-1* dsRNA, Figure 1C). Although similar phenotypes were observed when RNAi by feeding was performed, the penetrance of defects in the latter case was slightly lower (a 15% reduction in the case of *sft-1* feeding RNAi and a 43% reduction in the case of *oxa-1* feeding RNAi). In subsequent experiments, RNAi by injection was utilized as the more reliable method of choice.

*sft-1(RNAi)* animals developed at the same rate as WT N2 controls and did not exhibit embryonic lethality to any significant level, suggesting that the reduction in brood size is a consequence of reduced germ cell production. A similar reduction in brood size was noted for the F1 progeny of worms injected with *sft-1* dsRNA as for the injected mothers themselves. F2 progeny of injected mothers, as expected, had normal brood sizes (data not shown). The brood size of *sft-1(RNAi*) animals could be increased if F1 progeny from *sft-1* RNAi injected worms (at the L4 stage) were mated with WT males (data not shown), suggesting that defects in spermatogenesis are partly responsible for the lower brood size. However, supplying WT sperm did not restore brood sizes to normal, indicating that knockdown of *sft-1* leads to defects in both sperm and oocyte production.

*oxa-1* RNAi resulted in a high proportion of embryonic lethality (around 17% of the progeny of *oxa-1* dsRNA injected animals, Figure 1C). Dead eggs showed a range of defects at various stages in embryonic development, including regions of necrotic death in gastrulating early embryos, posterior abnormalities and failure to elongate further than the 1.5-fold ‘comma’ stage (Figure 1D). Viable offspring of injected mothers displayed a very pronounced slow growth (Gro) phenotype compared to N2 controls, taking over twice as long to reach adulthood, (for N2 controls, 100% of hatchlings reached L4 (by the criteria of a visible vulva) after 48 hours. For *oxa-1(RNAi)* animals, 0% of hatchlings reached L4 by 48 or 72 hours, and typically only 50% of hatchlings reached L4 (or at least some aspects of L4) after 96 hours). F1 progeny of *oxa-1(RNAi)* animals produced almost no progeny of their own.

### Increased lifespan in *sft-1(RNAi)* and *oxa-1(RNAi)* worms

**Figure 2:**
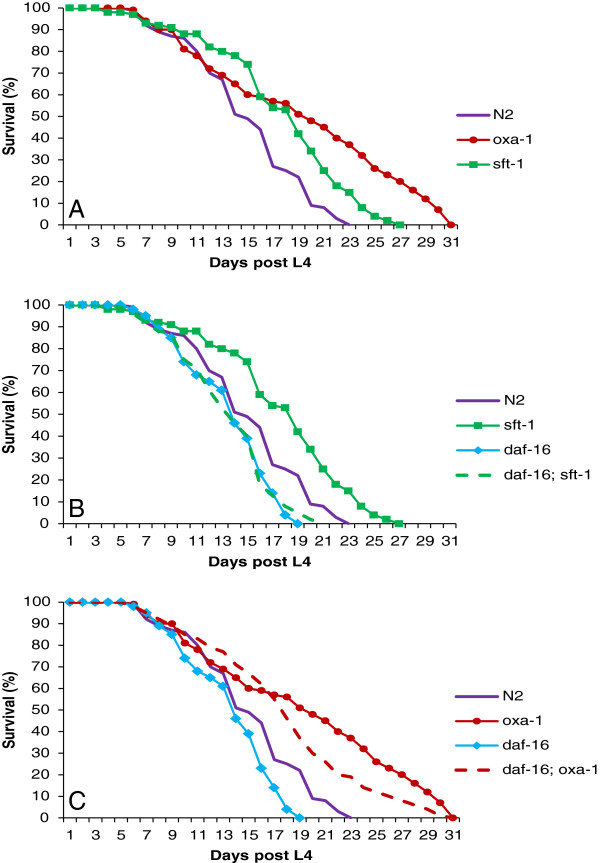
**Lifespan extension in *sft-1(RNAi) *and *oxa-1(RNAi) *animals. A**: Lifespan curves for N2, *oxa-1(RNAi)* and *sft-1(RNAi)* animals (recorded as number of days of survival post L4, or the time at which controls reached L4, in the case of *oxa-1(RNAi)* animals). Lifespan is prolonged in *sft-1(RNAi)* and *oxa-1(RNAi)* animals compared with WT controls, with *oxa-1* having the greatest effect. **B**: Lifespan analysis of *sft-1(RNAi)* animals performed in a *daf-16(m26)* mutant background, in which *sft-1* RNAi fails to extend lifespan. **C**: Lifespan analysis of *oxa-1(RNAi)* animals performed in a *daf-16(m26)* mutant background. *oxa-1* RNAi still extends lifespan under these circumstances. The data presented combine three independent data sets (the data for individual replicate experiments can be found in Additional file 1).

Cumulative survival curves of the F1 offspring of injected *sft-1(RNAi)* worms show an extended lifespan compared with the WT control (Figure 2A). Mean lifespan from L4 was 17.7 ± 0.6 days for *sft-1(RNAi)* worms (n = 95) and 15.1 ± 0.5 days for N2 control (n = 79), with 50% survival at days 19 and 15, respectively and maximal lifespans of 27 days for *sft-1(RNAi)* animals and 23 days for WT (the whole data set is shown in Additional file 1). With the F1 offspring of injected *oxa-1(RNAi)* worms, the lifespan was extended even further with a mean of 19.3 ± 0.8 days (n = 88), a maximum of 31 days and 50% survival at Day 20 (Figure 2A, Additional file 1). Experiments were begun with animals at the L4 stage, although it was difficult to precisely stage *oxa-1(RNAi)* animals given the developmental delay. It is possible that further developmental delay (that is, in progressing from L4 to adulthood) could contribute to the lifespan extension, although given that *oxa-1(RNAi)* animals, on average, took twice as long to reach adulthood, the delay from L4 to adulthood would only be expected to contribute to lifespan extension by a maximum of one day, and observed lifespan extension was much more pronounced than this.

To ascertain whether the lifespan extension was dependent on the *daf-16* insulin-like signaling pathway, RNAi of *sft-1* and *oxa-1* by injection was performed in a *daf-16*(*m26*) mutant background, which has previously been shown to suppress the lifespan extension of both *daf-2(e1370)* and *age-1(hx546)* mutants [28,29]. In a *daf-16(m26)* mutant background, *sft-1* RNAi resulted in mean and maximum lifespans of 13.5 ± 0.4 days (n = 92) and 21 days respectively, very similar to *daf-16* controls (13.6 ± 0.4 days (n = 92), maximum 19 days) (Figure 2B, Additional file 1). This suggests that lifespan extension in *sft-1(RNAi)* worms is dependent upon the *daf-16* pathway, and that *sft-1* may operate upstream of *daf-16* to regulate longevity.

In the case of *oxa-1* RNAi in a *daf-16* mutant background, however, a different result was obtained. In this case, lifespan was still extended following *oxa-1* knockdown, with a mean lifespan of 17.9 ± 0.7 days (n = 84) and maximum lifespan of 31 days, not statistically different to the data obtained when *oxa-1* RNAi is performed in a wild type background (Figure 2C, Additional file 1). This suggests that lifespan extension caused by *oxa-1* knockdown is independent of the *daf-16* insulin-like signaling pathway. However, we do note that the shape of the lifespan curves is somewhat different when *oxa-1(RNAi)* animals and *daf-16; oxa-1(RNAi)* animals are compared, and that the 50% survival point is reduced from 20 to 17 days, respectively. This may suggest a somewhat complex interaction between *oxa-1* and *daf-16*, or that suppression is incomplete with the *daf-16* allele used in this analysis, and caution needs to be exercised in the interpretation of this result. What is clear, however, is that the two knock-downs produce different phenotypes in terms of embryonic lethality, brood size and developmental delay, and respond somewhat differently to perturbations in the *daf-16* pathway, with *sft-1* showing complete dependence on functioning *daf-16* for lifespan extension, and the *oxa-1(RNAi)* phenotype being at least partially *daf-16*-independent.

It has previously been reported that, in the case of genes involved in mitochondrial function, the dose of dsRNA delivered to *C. elegans* in RNAi feeding experiments may affect the phenotypic outcome, with lower doses of particular dsRNAs causing lifespan extension while higher doses shorten lifespan [9]. It is well established that RNAi by injection tends to give more severe phenotypes than RNAi by feeding. In our experiments, we used undiluted dsRNA for injections, presumably representing the highest possible dose of RNAi. In addition, feeding RNAi experiments also resulted in lifespan extension (SM and AW, unpublished observations), suggesting that in the case of *sft-1* and *oxa-1*, the phenotypic outcome is unlikely to be dose-dependent (at least in terms of different doses resulting in opposite phenotypes).

### Response to oxidative stress following *sft-1* and *oxa-1* knockdown

To investigate whether the lifespan extension was associated with resistance to oxidative stress, *sft-1(RNAi)* and *oxa-1(RNAi)* worms were subjected to oxidative stress treatment with the herbicide paraquat. This causes oxidative stress by a metabolically catalyzed reaction resulting in depletion of NADPH and production of ROS, primarily superoxide anions [30].

**Figure 3:**
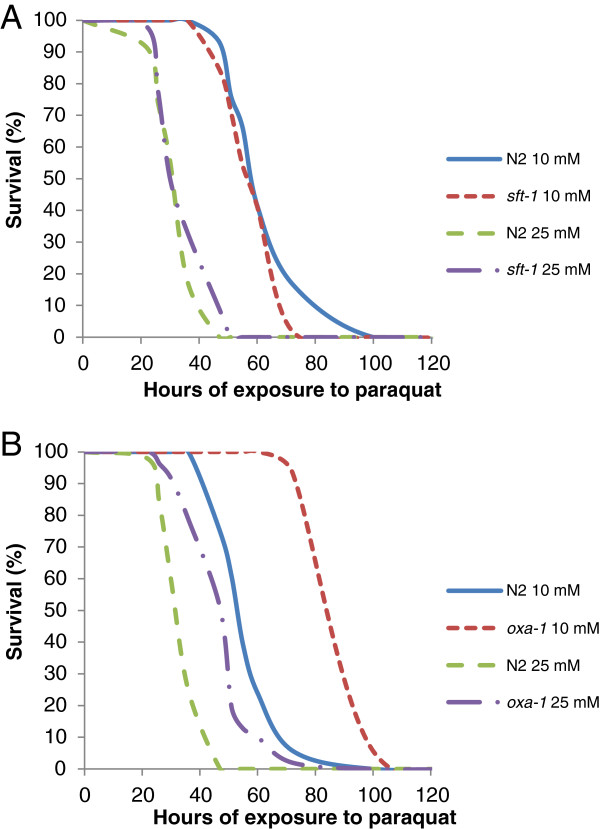
**Sensitivity of *sft-1(RNAi*) and *oxa-1(RNAi) *animals to oxidative stress. A**: Survival curves of WT or *sft-1(RNAi)* animals exposed to 10 mM or 25 mM paraquat, recorded as the number of hours of survival following exposure. WT and *sft-1(RNAi)* animals display similar sensitivity to paraquat at either concentration. **B**: Survival curves of size-matched WT and *oxa-1(RNAi)* animals exposed to paraquat. *oxa-1(RNAi)* animals show marked resistance to paraquat, at both 10 mM and 25 mM concentrations.

The resistance to oxidative stress of the *sft-1(RNAi)* worms was not significantly different from the N2 controls at either 10 or 25 mM paraquat (Figure 3A), with mean survival time following exposure of *sft-1(RNAi)* animals to 10 mM paraquat of 61.3 ± 2 hours (n = 36), compared to 65 ± 3.1 hours for WT controls (n = 29). Similarly, exposure to 25 mM paraquat resulted in a mean survival time of 35.4 ± 1.5 hours for *sft-1(RNAi)* animals (n = 36) compared with 33.8 ± 1.3 hours for WT controls (n = 35) (Additional file 2). By contrast, the *oxa-1(RNAi)* worms were much more resistant to paraquat than the control worms at both concentrations (Figure 3B). Given the Gro phenotype of *oxa-1(RNAi)* animals and, therefore, the time it takes for them to reach L4 (when our paraquat survival assays normally start), we used three-day-old progeny of *oxa-1* dsRNA injected hermaphrodites, and size-matched WT controls (L1s), to control for the possibility that animal size could have an effect on paraquat sensitivity that could complicate our results. In this case, exposure to 10 mM paraquat resulted in a mean survival time of 58.4 ± 1.9 hours for WT (n = 38) and 100.1 ± 1.9 hours for *oxa-1(RNAi)* animals (n = 42). Increasing the concentration of paraquat to 25 mM gave similar results, with a mean survival time of 35.9 ± 1.2 hours for WT (n = 39) and 49.7 ± 1.9 hours for *oxa-1(RNAi)* animals (n = 48) (whole data set shown in Additional file 3). In the case of the *oxa-1(RNAi)* animals used for this experiment, it is difficult to categorically state what developmental stage they were at. They were size-matched to L1 control WT animals, but may have been more advanced in terms of certain aspects of development, which could be uncoupled when *oxa-1* is knocked down and overall growth slows. In any case, however, it is noteworthy that WT animals did not appear to display any size-dependent resistance to paraquat (mean survival time of 65 ± 3.1 hours for L4 animals following exposure to 10 mM paraquat, compared with 58.4 ± 1.9 for L1s, and 33.8 ±1.3 hours for L4s compared with 35.9 ± 1.2 hours for L1s exposed to 25 mM paraquat), indicating that animal size differences are unlikely to be confounding our data.

### OXA-1 and SFT-1 tissue distributions

#### oxa-1::GFP

**Figure 4:**
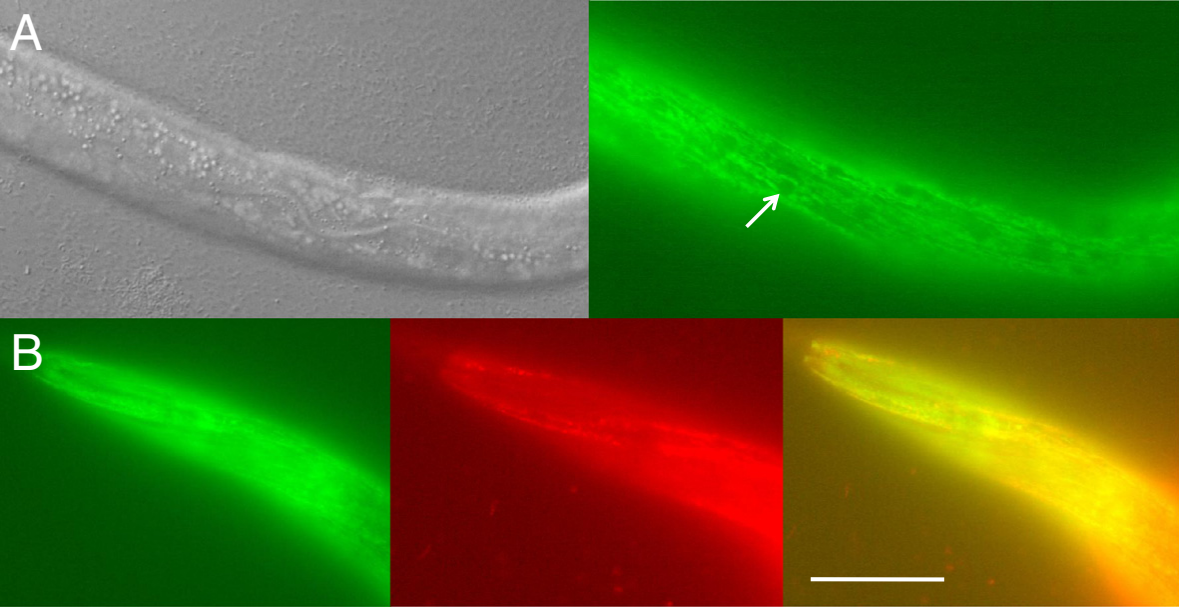
***oxa-1::gfp *expression pattern. A, B**: Transgenic strain *AW241* (*ouEx609 (pAW318 + rol-6*^*-*^*))*, where *pAW318* is a translational GFP fusion of *oxa-1* driven by the endogenous promoter. *oxa-1* is expressed at a high level throughout the worm in a reticular mitochondrial network pattern characteristic of mitochondrial proteins. A: expression of *oxa-1::gfp* is particularly strong in body wall muscle cells, where it is excluded from the nuclei (white arrow). B: co-localization of *oxa-1::gfp* (left hand green image) and Mitotracker Red (central red image) in the merged image (right hand yellow image), confirming expression of *oxa-1::gfp* in mitochondrial networks. Scale bar, 100 μm. Anterior is to the left in all images.

The expression pattern of a translational *oxa-1::gfp* fusion construct is shown in Figure 4. *oxa-1::gfp* is expressed at high levels throughout the animal. *oxa-1::gfp* expression is widely distributed, showing a punctate mitochondrial network pattern, and is particularly prominent in pharyngeal and body wall muscle (Figure 4). Mitochondria form elongated filaments or networks in many cell types that is particularly prominent in tissues with a high energy demand, such as muscle. To demonstrate that the expressed product did indeed localize to mitochondria, *oxa-1::gfp* worms were stained with Mitotracker Red and extensive co-localization was observed (Figure 4B). Embryonic *oxa-1::gfp* expression is very faint, but observable at the 100 cell stage (data not shown).

#### sft-1::gfp

The *sft-1* gene appears to be contained within a large operon, CEOP3088, making the construction of reporter constructs problematic, as it is difficult to predict where the important regulatory elements will be and the required constructs are likely to be very large (CEOP3088 covers some 40 Kb). Recent analysis suggests that *sft-1* is part of a “hybrid” operon, however, containing intercistronic promoter/enhancer elements required for tissue-specific gene expression [31]. Given the relatively large intercistronic region in the case of *sft-1* (>1.5 Kb), we made a translational reporter construct in which this region was used to drive *sft-1::gfp*, which would be expected to report on at least part of the *sft-1* expression pattern. The expression pattern obtained with this construct is shown in Additional file 4. *sft-1::GFP* is expressed at a very low level throughout the worm with higher levels of expression in a region of body wall muscle adjacent to the pharyngeal bulb (Additional file 4A) and the uterine area (Additional file 4B). The low overall level of expression may well reflect the absence of operon-upstream regulatory elements from the *sft-1::gfp* construct.

## Discussion

Cytochrome oxidase deficiency generated by RNAi of *sft-1* resulted in lifespan extension accompanied by decreased germ cell production but no obvious slowdown of developmental rate, whereas the lifespan extension caused by RNAi of *oxa-1* was associated with a pronounced slow growth phenotype and lethal defects in embryonic development. The combination of dead embryos and long-lived progeny in the broods of *oxa-1(RNAi)* animals could reflect some mitochondrial “threshold effect” in which the threshold for mitochondrial function is surpassed in some embryos, leading to catastrophic mitochondrial damage and the inability to invoke compensatory pathways.

The embryonic lethality and slow growth of viable progeny observed with RNAi of *oxa-1* correlates with phenotypic observations of *oxa-1* homologues in other organisms. Inactivation of both *OXA1* genes in *S. pombe* is lethal [32], whereas a knockout mutant of *S. cerevisiae* is viable, but is unable to respire and can only grow on fermentable carbon sources [4]. The function of these various OXA1 proteins is conserved, as *N. crassa*, human and *S. pombe* homologues can rescue *S. cerevisiae oxa1* null mutants [33]. In yeast, defects in *Oxa1* ablate the function of the cytochrome oxidase complex and greatly reduce levels of Complex III and F_0_F_1_ATPase [34]. Overall, the data suggest that OXA1 is important for the biogenesis of several different respiratory chain complexes (reviewed in [35]) and this may account for the severity of the *oxa-1* RNAi phenotype seen in *C. elegans*. Consistent with this, no *OXA1* deficient patients have been identified and this condition may thus be embryonic lethal in humans.

*SURF1* deficient patients, on the other hand, usually survive into early childhood with a progressive neurodegenerative disease. In contrast to the human SURF1 deficient phenotype, no abnormal neuromuscular function was observed in *sft-1*(RNAi) worms, despite detailed observations of movement and pharyngeal pumping rates (data not shown). A possible caveat to this conclusion, however, is that RNAi in *C. elegans* is known to be relatively ineffective in the nervous system, thus neuronal phenotypes may have been masked in our experiments. It is also possible that the lack of a neuromuscular phenotype in the *sft-1* deficient worms may simply reflect overall energy requirements and the level of energy generation in different tissues. Previous studies of metabolic activity in *C. elegans*, as monitored by oxygen consumption, indicate that only a small proportion of total energy expenditure is on movement and the greatest single energetic demand is for reproduction [36]. There is a significant increase in oxygen consumption at the L3 and L4 larval stages, the time of extensive proliferation of germ cells (reviewed in [37]. Reflecting this dependence of reproduction on energy generating capacity, mutations leading to deficiency of two other components of the mitochondrial oxidative phosphorylation system, Complex I (*nuo-1* gene) and the F_1_F_0_ ATP synthase (*atp-2* gene) also show a marked abnormality in gonadal development [13].

Decreased fecundity of *sft-1*(RNAi) worms was attributed to a failure of both oogenesis and spermatogenesis, as unfertilized oocytes were not observed at a significant level and WT sperm was only able to partially restore the brood size. Consistent with the hypothesis that decreased energy generating capacity in *sft-1*(RNAi) worms due to COX deficiency may account for the reproductive failure, we observed high levels of COX activity in the germ line of WT animals. *sft-1::gfp* was not observed in the germline, but a likely explanation for this is the phenomenon of gene silencing that is often triggered by transgene expression in the germline [38].

Both *sft-1(RNAi)* and *oxa-1(RNAi)* worms can be described as having Mit phenotypes (that is, those associated with mitochondrial dysfunction). Knockdown of either gene resulted in a significantly extended lifespan (particularly in the case of *oxa-1*) in at least two independent experiments, while only the *oxa-1(RNAi)* worms were resistant to oxidative stress induced by treatment with paraquat. The relationship between mitochondrial function and lifespan in *C. elegans* is complex, although most observations support two contributory mechanisms, nutritional restriction and damage due to ROS. Many different observations link nutritional/energetic status and lifespan in a wide range of different species. Food deprivation, either environmental or genetically determined (for example, *eat-2* gene mutations [39]) and mutations in genes involved directly (for example, *nuo-1*[13]) or indirectly (for example, *clk-1*[40]) in mitochondrial energy metabolism have all been shown to prolong life in *C. elegans*. These observations are incorporated in the “rate of living model” for longevity in which increased life expectancy is attributed to a generalized slowing down of metabolic function.

In nearly all cases examined thus far, lifespan extension in Mit mutants has been shown to act independently of the insulin/IGF signaling pathway [5]. Therefore, the dependence of *sft-1(RNAi)* animals on *daf-16* for lifespan extension, suggesting that *sft-1* may act upstream of *daf-16* to regulate longevity, is noteworthy, putting *sft-1* knockdown in a different phenotypic category from most other Mit phenotypes. The FoxO-like forkhead/winged helix transcription factor DAF-16 is thought to be a master regulator of aging, integrating metabolic signals, stress signals as well as reproductive signals from the germline to modulate longevity [41]. Perhaps the dependence of lifespan extension in *sft-1(RNAi)* animals on *daf-16* reflects the relative importance of reduced fecundity in promoting longevity when *sft-1* expression is reduced.

The second model for the determination of lifespan in *C. elegans* is based on the accumulation of damage due to ROS, although recent analyses suggest a rather complex relationship between oxidative stress and aging, and one that can be experimentally uncoupled. The oxidative damage model is not independent of the rate of living model as the major site for the formation of ROS is the mitochondrion and production is related to the level of metabolic activity. Some Mit mutants (for example, *lrs-2* and *isp-1*), in common with IGF/insulin signaling pathway mutants *daf-2* and *age-1*, show increased lifespan and resistance to oxidative stress [12], reviewed in [8]. *oxa-1*, as presented here, appears to fall into this category. By contrast, *gas-1*, *nuo-1* and *mev-1* mutations confer reduced lifespan associated with a hypersensitivity to free radicals [16,42,43], reviewed in [8]. Resistance to oxidative stress is suggested to extend lifespan by decreasing damage caused by accumulation of free radicals in the cell.

On the other hand, mutants such as *clk-1* and *sod-2* display a hypersensitivity to oxidative stress but are also long-lived [18,22], suggesting that oxidative stress *per se* is not always a primary cause of aging. Indeed, it has been suggested that ROS generated inappropriately in some mitochondrial mutants might actually extend lifespan by signaling various adaptive responses [12,44]. This proposed preconditioning of mitochondria has been termed mitohormesis [45]. Intriguingly, though, there do not appear to be mutants that are highly resistant to oxidative stress yet display a shortened lifespan, supporting the idea that oxidative stress resistance (or adaptation to oxidative stress) is associated with a long life.

“Oxidative stress resistance” is rather an umbrella term, as different Mit mutants display different sensitivities to a spectrum of oxidative stresses. For example, in one study of 10 different mutant/RNAi strains, most of the long-lived worms with compromised mitochondria displayed marked resistance to hydrogen peroxide, yet were not resistant (or even displayed increased sensitivity) to paraquat [12]. The authors of this study proposed that as paraquat triggers the production of superoxide in a NADPH-dependent reaction, paraquat hypersensitivity of worms with mitochondrial dysfunction might be due to an increase in NADPH associated with a particular metabolic response to reduced electron transport. However, the data that we present here indicate that *oxa-1(RNAi)* worms, in contrast to those analyzed in the Lee study, exhibit marked resistance to paraquat. This suggests that it is very difficult to generalize about the effects of mitochondrial dysfunction, even within the group of mutants/RNAi-treated worms that display both prolonged lifespan and heightened stress resistance. Furthermore, a recent study has found that pathways of lifespan extension can differ depending on whether RNAi or mutation for the same gene is used to reduce gene function [46]. Mutations in *sft-1* or *oxa-1* were not analyzed here, but these would form an interesting subject for future analysis.

It has been previously suggested that long-lived Mit mutants utilize a novel metabolism, and that longevity in these animals may be dependent on this altered metabolic state. For example, it has been proposed, albeit using a limited number of mutants, that long-lived Mit mutants up-regulate fermentative malate dismutation, where fumarate is terminally reduced at complex II to succinate, generating fewer radical species overall [17]. This is an anaerobic metabolic pathway thought to be unique to nematodes, and normally up-regulated during dauer formation. However, other studies argue that although metabolic restructuring does indeed occur in Mit mutants, the restructuring *per se* does not cause lifespan extension [47]. Recent data, however, challenge this view. For example, the importance of the alternative glyoxylate pathway in Mit mutant lifespan extension has been investigated by knocking down *gei-7,* which encodes the main glyoxylate shunt enzyme in *C. elegans*. *gei-7* mutation was found to suppress the enhanced longevity of *clk-1* mutants [48] and has also been found to reduce the lifespan of *cyc-1* Mit mutants [49].

Strikingly, mitochondrial respiratory complex dysfunction models being developed in other systems display many of the same features as *C. elegans* Mit mutants. For example, a partial deficiency of *Mclk1*, the mouse *clk-1* ortholog, increases average lifespan by 30% [50]. Even more relevant to this study, increased lifespan following inactivation of *Surf1* in mice has been recently demonstrated [51], and similarly, *Surf1* knockdown in the central nervous system of *Drosophila melanogaster* has also been shown to induce longevity [52]. This suggests that the function of *sft-1* in regulating lifespan is likely to be widely conserved, and it will be very interesting to discover the extent to which precise mechanisms of lifespan extension are conserved between disparate species. For example, it would be interesting to examine whether lifespan extension in *sft-1* knockdown animals is dependent on *gei-7* and thus a shift to the glyoxylate pathway. It is not clear, however, how such metabolic restructuring might proceed in other organisms where this alternative pathway is not thought to operate.

Whatever the precise mechanisms, it is clear that *sft-1* and *oxa-1* influence longevity through distinct molecular pathways, despite the fact that both genes encode factors required for COX assembly.

## Conclusions

We have clearly shown that knockdown of *sft-1* or *oxa-1* by RNAi results in increased longevity; however, distinct molecular mechanisms appear to operate in each case, and analysis of these two genes illustrate the principle that it is not possible to draw simple conclusions about the relationships between mitochondrial energetics, longevity, stress resistance and insulin-like signaling. Further analysis of COX deficiency and generation of worms with defects in other mitochondrial enzyme complexes will be required to further delineate the organismal consequences of mitochondrial dysfunction that will help provide new models for human mitochondrial-associated diseases. For the present, analysis of the effects of reducing expression of *sft-1* and *oxa-1*, both of which encode proteins required for the assembly of respiratory complexes, illustrates the diversity of phenotypic outcomes that can result from mitochondrial dysfunction and the complex inter-relationships that exist between energy metabolism, reproduction and aging even in this simplest of metazoan model organisms.

## Methods

### *C. elegans* strains and maintenance

Experiments were performed with the wild-type (WT) Bristol strain N2 and *daf-16(m26)* mutant (provided by the Caenorhabditis Genetics Center). *C. elegans* strains were maintained and handled on nematode growth media (NGM) agar with *E. coli OP50* as their food source [52]. Microscopy was carried out using a Zeiss Axiophot microscope (Carl Zeiss Ltd, Bicester, Oxon, UK) fitted with DIC and fluorescence optics as appropriate. Worms were anesthetized on 2% agarose pads as previously described [53].

### Bioinformatic analysis

Homologs were identified by BLAST analysis (http://blast.ncbi.nlm.nih.gov). *C. elegans* protein sequences were retrieved from Wormbase (http://www.wormbase.org) and human protein sequences from the Ensembl database (http://www.ensembl.org). Sequences were analyzed using Pairwise alignment in Bioedit (http://www.mbio.ncsu.edu/bioedit/bioedit.html).

### RNAi by injection

PCR primers specific for *sft-1* (*C. elegans* gene *H06I04.2*) and *oxa-1* (*C01A2.3)* and including T7 or T3 RNA polymerase promoter sites were designed. These amplified fragments of 679 bp and 515 bp were from genomic DNA, respectively.

*sft-1* F primer 5’ ATTAACCCTCACTAAAGTTATTTTGAAGC 3’ JH1;

*sft-1* R primer 5’AATACGACTCACTATAGGTGACGGGGAATTC 3’ JH2

*oxa-1* F primer 5’ATTAACCCTCACTAAAGACATTCCCTGGTGGGTTACA 3’ JH5.

*oxa-1* R primer 5’AATACGACTCACTATAGACGCATAATTGGTGGCATTT 3’ JH6

dsRNA was synthesized directly from PCR products as previously described [54] and injected into young adult hermaphrodites as previously described [55]. Injected worms were transferred to fresh seeded plates four hours after injection and the progeny assayed for relevant phenotypes, except in the case of brood size measurements, which were performed on the injected mothers themselves as well as the subsequent F1 progeny.

### RNAi by feeding

L3 N2 (or *daf-16(m26)*) hermaphrodites were picked onto IPTG plates which had been seeded with the relevant bacterial RNAi feeding clones as previously described [56]. The *oxa-1* feeding clone in the feeding RNAi bacterial strain *HT115* was from the Ahringer Lab RNAi feeding library [57]. The *sft-1* feeding clone was made by inserting a PCR fragment corresponding to 400 bp of *sft-1* exonic sequence (amplified from fosmid H06104) into the L4440 RNAi feeding vector, using enzymes *XbaI* and *HindIIII.* This was electroporated into *E. coli* strain HT115 before feeding to worms.

### RT-PCR

Reduction of the target transcripts following RNAi treatment was confirmed by gene-specific RT-PCR using the Superscript III RT system (Life Technologies (Invitrogen Division). Renfrew, Paisley, UK) with RNA from 20 L4 progeny from *sft-1(RNAi)* worms and six plates of progeny from *oxa-1(RNAi)* worms and equivalent N2 controls. Transcript levels of a non-target gene, *eft-2*, were measured as a control for the specificity of the RNAi. The RT reaction was primed using oligo(dT) primers. RNA was extracted using a hot phenol protocol [58] and purified using a DNAse column. A total of 5 μL of RNA dissolved in RNAse free water was used in the RT reaction to synthesize cDNA.

Specific *sft-1*, *oxa-1*and *eft-2* RT-PCR primers were designed and amplified 220 bp, 164 bp and 350 bp fragments, respectively. Twenty-five cycles of PCR were used in each case.

*sft-1* F primer 5’ GAAAGGGCGACTGAATCAAA 3’ JH7

*sft-1* R primer 5’ GTGTCCTCCATGACTGCTCA 3’ JH8

*oxa-1* F primer 5’ GCACTTCCATTCATCTCTGC 3’ JH9

*oxa-1* R primer 5’ CATAGACCCGTAGCAAATTGTG 3’ JH10

*eft-2* F primer 5’ GCGTATCAAGCCAGTTCTTT 3’ JH19

*eft-2* R primer 5’ CTGCTCCACTTCTTGGTCTT 3’ JH20

### Brood counts

Brood sizes were assessed using 20 synchronized L4 worms each injected with dsRNA corresponding to *sft-1* or *oxa-1*. Control groups were injected with TE buffer only. (In the case of feeding RNAi experiments, L3 animals were first picked onto NGM plates seeded with HT115 bacteria transformed with the relevant feeding RNAi clone or empty vector control). For the brood size counts, L4 animals were picked singly onto individual 55 mm NGM plates seeded with OP50 bacteria (or HT115 transformed with the relevant feeding RNAi clone or empty vector control), and transferred to fresh seeded plates every 24 hours until egg laying had stopped. Counting of unhatched eggs and live progeny was performed on plates from which the mother had been moved the previous day and individual plate counts totaled in order to derive a complete brood size for each animal. All plates were kept at 20°C.

### Cytochrome oxidase (COX) staining

COX activity was assayed in cells of intact worms by the oxidation of diaminobenzidine in the presence of cytochrome c in a protocol adapted from Seligman *et al. *[59]. The specificity of the COX staining reaction was demonstrated by the absence of reaction product when worms were incubated with sodium azide (NaN_3_). A total of 50 mixed-stage animals (either the progeny of animals injected with *sft-1* dsRNA, or controls) were picked into 50 μl of M9 buffer in an Eppendorf tube (in duplicate) (Eppendorf UK Ltd, Stevenage, Herts, UK). The worms were allowed to settle and the M9 buffer was carefully removed. A total of 1 ml of freshly prepared COX stain (1 mg/mL diaminobenzidine-HCl, 1 mg/mL cytochrome c, 2 mg/mL catalase in 50 mM sodium phosphate buffer, pH 7.4) was added to the worms and gently mixed. Worms were incubated with the stain for 4 h at 20°C. NaN_3_ was added to one of the duplicate tubes to a final concentration of 1 mM. The COX stain was removed and the worms gently rinsed with PBS twice. Tubes were spun at 6,000 rpm for 8 secs to gently sediment the worms between rinses. The worms were then placed on a slide and allowed to air dry (approximately 30 minutes). They were subsequently mounted for microscopy by adding a coverslip coated with Aquatex (VWR International Ltd, Lutterworth, Leics, UK) and samples photographed using identical settings and exposure times. Experiments were repeated at least three times.

### Determination of lifespan

Lifespan assays were performed using 20 to 50 worms for each strain (or F1 progeny of animals that had been injected with dsRNA corresponding to *sft-1* or *oxa-1*, or fed on the appropriate feeding RNAi bacteria) per experiment. Single L4 worms were picked onto individual wells of 12-well NGM plates seeded with OP50 bacteria, and transferred daily to a fresh seeded well until the end of egg laying. Animals were checked for viability every day by recording their response to mechanical stimulus. Worms that did not respond to five gentle prods with a platinum wire were scored as dead and the date of death recorded. All lifespan assays were performed at 20°C, with three biological replicates.

### Sensitivity to oxidative stress

Oxidative stress sensitivity assays were performed using 50 worms for each strain (or F1 progeny of animals that had been injected with dsRNA corresponding to *sft-1* or *oxa-1*).

*sft-1(RNAi)* or *oxa-1(RNAi)* worms and size-matched N2 controls were picked onto 30 mm NGM plates (seeded with OP50 bacteria, 10 animals per plate) containing final concentrations of 0, 10 or 25 mM paraquat (1,1'-Dimethyl-4,4'-bipyridinium dichloride hydrate, Sigma (Sigma-Aldrich, Poole, Dorset, UK)). Survival was assessed at regular intervals over the course of 100 hours by response to mechanical stimulus as described above. Animals that escaped from the plate and thus could not be accounted for were excluded from the analysis. All plates were kept at 20°C.

### *sft-1* and *oxa-1* GFP reporter constructs

*sft-1* and *oxa-1* translational GFP fusion constructs were made using the PCR fusion method [60]. An 8,129 bp fragment containing the *sft-1* ORF (H06I04.2) and 3,204 bp of upstream sequence was amplified from fosmid H06I04 using Expand Long Template polymerase (Roche Diagnostics Ltd., Burgess Hill, West Susses, UK) and primers JH13 and JH15. JH15 includes a tag complementary to sequence at the 5’ end of GFP.

*sft-1* F primer 5’GTAAGCTGGAATCGGCAAAG 3’ JH13

*sft-1* R primer 5’ CGACCTGCAGGCATGCAAGCTCCACATGAGCATTGTGAC 3’ JH15.

A 6,870 bp fragment containing the *oxa-1* ORF (C01A2.3) and 3,770 bp of upstream sequence (including all putative regulatory sequence elements within the operon CE0P1696) was amplified from fosmid I6F08, using Expand Long polymerase and primers JH16 and JH18. JH18 includes a tag complementary to sequence at the 5’ end of GFP.

*oxa-1* F primer 5’ CGTCTCTCCCATCCAGCTT 3’ JH16

*oxa-1* R primer 5’ CGACCTGCAGGCATGCAAGCATGAAGCATCACGTTGTCG 3’ JH18

To fuse the gene specific PCR products to the GFP reporter, 0.5 μL of each PCR product and 0.5 μL of gel purified GFP PCR product (1.8 kb fragment, the product of PCR with primers ppdgfp and gfp c1 from plasmid pPD95.75 (Fire Lab vectors obtained from Addgene, Cambridge, MA, USA) were added to the Expand Long Buffer 3 system reaction mix to form the template for the sewing reaction. A second forward nested primer and GFP specific reverse primer (gfp c2) were used to amplify the full-length gene-GFP fused PCR product. These products were gel purified using the SYBR-RED gel purification system.

*sft-1* nested forward primer: 5’GTGTATGCAAATGCGACGAG 3’ JH14

*oxa-1* nested forward primer 5’CGTCTCTCCCATCCAGCTT 3’ JH17

ppdgfp primer 5’GCTTGCATGCCTGCAGGTCG3’

gfp c1 primer 5’AAGGGCCCGTACGGCCGACTAGTAGG3’

gfp c2 primer 5’AAACAGTTATGTTTGGTATATTGGG3’

The purified PCR products were cloned into the TOPO XL vector using the TOPO XL cloning kit (Invitrogen) and shown to contain the correct inserts by restriction digest and sequencing. The *sft-1::gfp* construct is pAW317 and the *oxa-1::gfp* construct is pAW318.

### Transgenic worms

GFP reporter constructs were injected into the syncytial gonad of young adult hermaphrodite worms at a concentration of approximately 20 ng/μL as described [55] along with the *rol-6*^*-*^ transformation marker *pCes1943*. Rol progeny were picked and stable lines selected for analysis. The *sft-1::gfp* transgenic strain described in this study is AW240 (*ouEx608 (pAW317 + rol-6*^*-*^*))* and the *oxa-1::gfp* transgenic strain is AW241 (*ouEx609 (pAW318 + rol-6*^*-*^*))*.

### Mitotracker staining

L4 stage N2 and AW241 worms were transferred to seeded NGM plates with 2 μg/ml Mitotracker Red (Life Technologies Ltd (Invitrogen division, Renfrew, Paisley, UK) spread on the surface and stored in the dark. L4 progeny from these animals were picked, washed in M9 for one hour and mounted for fluorescence microscopy as described above.

## Abbreviations

COX: Cytochrome oxidase; MIT: Mitochondrial; NGM: Nematode growth media; PBS: Phosphate-buffered saline; ROS`: Reactive oxygen species.

## Competing interests

The authors declare that they have no competing interests.

## Authors’ contributions

SM, JH, CB and PA carried out the brood size, life span and oxidative stress assays. RB and CD performed the COX staining experiments, and SM, JH and RB constructed and analyzed the GFP expression patterns. GB and AW conceived of the study, participated in its design and coordination, and wrote the manuscript. All authors read and approved the final manuscript.

## Supplementary Material

Additional file 1 **Lifespan extension in *sft-1(RNAi)* and *oxa-1(RNAi) *** **worms displays differential dependence on the insulin-like signalling pathway. A**: Combined dataset for three separate experiments. Analytical values for lifespan experiments are shown, including mean and median adult lifespan, maximum adult lifespan and the sample size (*n*) for each strain and experimental condition. Statistical tests (Log-Rank tests using OASIS software [61]) were carried out using the lifespan of each worm in the entire population. *oxa-1(RNAi)* and *sft-1(RNAi)* animals have a significantly increased mean lifespan compared with the wild type N2 strain (*P* = 1 x 10^-7^ and 2 x 10^-5^, respectively). *daf-16(m26); oxa-1(RNAi)* animals have a significantly increased lifespan compared with *daf-16* alone (*p<* 1 x 10^-10^), but the difference is not significant when compared with *oxa-1* alone (*p* = 0.09). *daf-16(m26); sft-1(RNAi)* animals do not have a significantly different lifespan from *daf-16* alone (*P* = 0.75) but these animals have a significantly decreased lifespan compared with *sft-1(RNAi)* alone (*P <* 1 x 10^-10^). **B**: Individual datasets for the three separate experiments. Mean, median and maximum lifespans are shown. Log-Rank tests were carried out using the lifespan of each worm in the entire population. * denotes the significance (*P*) value compared to N2 worms, ** denotes the *P-*value compared to *daf-16(m26)* worms. *oxa-1(RNAi)* animals had a significant lifespan extension compared to control animals in all three biological replicates which was at least partially independent of *daf-16*. *sft-1(RNAi)* animals had a significant lifespan extension compared to control animals in two out of three biological replicates (in the third replicate the *P-*value was 0.079). In all cases, the lifespan extension was dependent on *daf-16* (that is, significant lifespan extension was not observed in a *daf-16(m26*) mutant background). Click here for file

Additional file 2 **Sensitivity of *sft-1(RNAi)* animals to oxidative stress.** Analytical values for survival following exposure to different concentrations of paraquat are shown, including mean and median survival (in hours), standard error of the mean (SEM), maximum survival and the sample size (n) for each strain and experimental condition. Statistical tests (*t*-tests) were carried out using the survival time of each worm in the population. *sft-1(RNAi)* animals do not have a significantly different mean survival time compared with N2 controls after exposure to either 10 mM or 25 mM paraquat (*P* = 0.30 and 0.41, respectively). Click here for file

Additional file 3 **Resistance of *oxa-1(RNAi) *** **animals to oxidative stress.** Analytical values for survival following exposure to different concentrations of paraquat are shown, including mean and median survival (in hours), standard error of the mean (SEM), maximum survival and the sample size (n) for each strain and experimental condition. Statistical tests (*t*-tests) were carried out using the survival time of each worm in the population. *oxa-1(RNAi)* animals displayed a significantly increased mean survival time compared with N2 controls following exposure to either 10 mM or 25 mM paraquat (*P* = 1 x 10^-25^ and 1 x 10^-7^, respectively). Click here for file

Additional file 4 ***sft-1::gfp *** **expression pattern. A, B**: Transgenic strain *AW240* (*ouEx608 (pAW317 + rol-6*^*-*^*))*, where *pAW317* is a translational GFP fusion of *sft-1* driven by the intercistronic promoter. *sft-1::gfp* is expressed at a very low level throughout the worm, but is concentrated in particular tissues. Left hand images, DIC only, right hand images, DIC and GFP merged. A: *sft-1::gfp* expression in muscle surrounding the pharynx (white arrow). B: *sft-1::gfp* expression in the uterus (white arrowheads). Scale bar, 100 μm. Anterior is to the left in all images. Click here for file
