# Supplementary material for: The SFT-1 and OXA-1 respiratory chain complex assembly factors influence lifespan by distinct mechanisms in C. elegans
Source: Longev Healthspan. 2013 May 8;2:9. doi: 10.1186/2046-2395-2-9 (PMC3922957; doi:10.1186/2046-2395-2-9)
Supplement: Additional file 1 — Lifespan extension in sft-1(RNAi) and oxa-1(RNAi) worms displays differential dependence on the insulin-like signalling pathway. A: Combined dataset for three separate experiments. Analytical values for lifespan experiments are shown, including mean and median adult lifespan, maximum adult lifespan and the sample size (n) for each strain and experimental condition. Statistical tests (Log-Rank tests using OASIS software [61]) were carried out using the lifespan of each worm in the entire population. oxa-1(RNAi) and sft-1(RNAi) animals have a significantly increased mean lifespan compared with the wild type N2 strain (P = 1 x 10^-7^ and 2 x 10^-5^, respectively). daf-16(m26); oxa-1(RNAi) animals have a significantly increased lifespan compared with daf-16 alone (p< 1 x 10^-10^), but the difference is not significant when compared with oxa-1 alone (p = 0.09). daf-16(m26); sft-1(RNAi) animals do not have a significantly different lifespan from daf-16 alone (P = 0.75) but these animals have a significantly decreased lifespan compared with sft-1(RNAi) alone (P < 1 x 10^-10^). B: Individual datasets for the three separate experiments. Mean, median and maximum lifespans are shown. Log-Rank tests were carried out using the lifespan of each worm in the entire population. * denotes the significance (P) value compared to N2 worms, ** denotes the P-value compared to daf-16(m26) worms. oxa-1(RNAi) animals had a significant lifespan extension compared to control animals in all three biological replicates which was at least partially independent of daf-16. sft-1(RNAi) animals had a significant lifespan extension compared to control animals in two out of three biological replicates (in the third replicate the P-value was 0.079). In all cases, the lifespan extension was dependent on daf-16 (that is, significant lifespan extension was not observed in a daf-16(m26) mutant background). [file 2046-2395-2-9-S1.docx]

Additional File 1

A.

| **Strain** | **Mean**  **(days**  **survival)** | **SEM** | **Median** | **Maximum** | **n** |
| --- | --- | --- | --- | --- | --- |
| N2  *oxa-1(RNAi)*  *sft-1(RNAi)*  *daf-16 (m26)*  *daf-16 (m26); oxa-1(RNAi)*  *daf-16 (m26); sft-1(RNAi)* | 15.1  19.3  17.7  13.6  17.9  13.5 | 0.5  0.8  0.6  0.4  0.7  0.4 | 15  20  19  14  18  14 | 23  31  27  19  31  21 | 79  88  95  92  84  92 |

B.

| **Strain** | **Mean**  **(days**  **survival)** | **SEM** | **Median** | **Maximum** | **n** | ***P* value**  **compared**  **with control** |
| --- | --- | --- | --- | --- | --- | --- |
| N2  experiment 1  2  3 | 15.1  15.4  14.7 | 0.7  1.0  1.0 | 14  16  15 | 22  23  22 | 37  22  20 | -  -  - |
| *oxa-1(RNAi)* experiment 1  2  3 | 20.3  19.3  17.8 | 1.4  1.4  1.5 | 22.5  20  16 | 31  31  30 | 36  28  24 | *  3 x 10^-5^  0.007  0.05 |
| *sft-1(RNAi)* experiment 1  2  3 | 18.2  17.5  17.1 | 0.7  1.2  1.1 | 19  19  19 | 27  25  26 | 46  22  27 | *  0.0006  0.079  0.03 |
| *daf-16(m26)* experiment 1  2  3 | 14.2  13.6  12.8 | 0.5  0.8  0.7 | 14.5  15  13 | 19  19  18 | 40  25  27 | *  0.03  0.056  0.05 |
| *daf-16(m26); oxa-1(RNAi)* experiment 1  2  3 | 19.1  17.7  16.6 | 0.9  1.1  1.4 | 19  17  17 | 31  29  30 | 30  29  25 | **  3 x 10^-7^  0.0007  0.0035 |
| *daf-16(m26); sft-1(RNAi)* experiment 1  2  3 | 14.1  13.9  12.5 | 0.4  0.8  0.9 | 15  14  12 | 18  21  20 | 39  25  28 | **  0.49  0.58  0.59 |
