# Supplementary material for: The SFT-1 and OXA-1 respiratory chain complex assembly factors influence lifespan by distinct mechanisms in C. elegans
Source: Longev Healthspan. 2013 May 8;2:9. doi: 10.1186/2046-2395-2-9 (PMC3922957; doi:10.1186/2046-2395-2-9)
Supplement: Additional file 3 — Resistance of oxa-1(RNAi) animals to oxidative stress. Analytical values for survival following exposure to different concentrations of paraquat are shown, including mean and median survival (in hours), standard error of the mean (SEM), maximum survival and the sample size (n) for each strain and experimental condition. Statistical tests (t-tests) were carried out using the survival time of each worm in the population. oxa-1(RNAi) animals displayed a significantly increased mean survival time compared with N2 controls following exposure to either 10 mM or 25 mM paraquat (P = 1 x 10^-25^ and 1 x 10^-7^, respectively). [file 2046-2395-2-9-S3.docx]

Additional File 3

| **Strain** | **Mean**  **(hours**  **survival)** | **SEM** | **Median** | **Maximum** | **n** |
| --- | --- | --- | --- | --- | --- |
| N2 10mM paraquat  *oxa-1(RNAi)* 10mM paraquat  N2 25mM paraquat  *oxa-1(RNAi)* 25mM paraquat | 58.4  100.1  35.9  49.7 | 1.9  1.9  1.2  1.9 | 55  100  36  51 | 100  144  47  100 | 38  42  39  48 |
