# Supplementary figures and images for: The SFT-1 and OXA-1 respiratory chain complex assembly factors influence lifespan by distinct mechanisms in C. elegans
Source: Longev Healthspan. 2013 May 8;2:9. doi: 10.1186/2046-2395-2-9 (PMC3922957; doi:10.1186/2046-2395-2-9)

## Slide 1
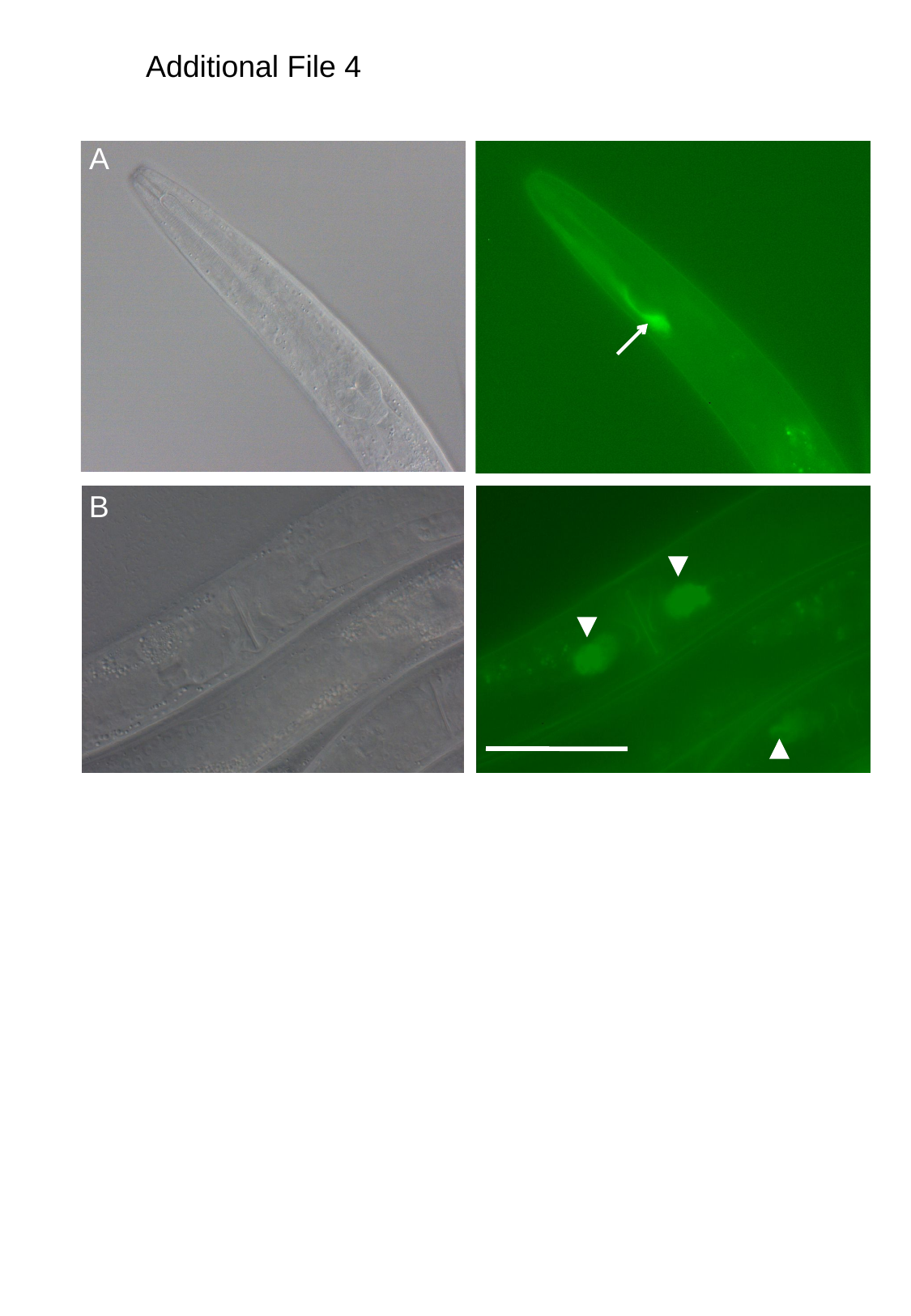

Additional File 4
A
B

Supplement: Additional file 4 — sft-1::gfp expression pattern. A, B: Transgenic strain AW240 (ouEx608 (pAW317 + rol-6 - )), where pAW317 is a translational GFP fusion of sft-1 driven by the intercistronic promoter. sft-1::gfp is expressed at a very low level throughout the worm, but is concentrated in particular tissues. Left hand images, DIC only, right hand images, DIC and GFP merged. A: sft-1::gfp expression in muscle surrounding the pharynx (white arrow). B: sft-1::gfp expression in the uterus (white arrowheads). Scale bar, 100 μm. Anterior is to the left in all images. [file 2046-2395-2-9-S4.ppt]
